# Neutrophil redox imbalance in acute coronary syndrome

**DOI:** 10.5937/jomb0-59659

**Published:** 2026-02-27

**Authors:** Ilija Dragojević, Dijana Mirić, Bojana Kisić, Dragana Puhalo-Sladoje, Ljiljana Popović, Dragiša Rašić

**Affiliations:** 1 University of Priština - Kosovska Mitrovica, Faculty of Medicine, Kosovska Mitrovica; 2 University of East Sarajevo, Faculty of Medicine Foča, Foča, Republic of Srpska, B&H

**Keywords:** acute coronary syndrome, neutrophils, oxidative stress, akutni koronarni sindrom, neutrofili, oksidativni stres

## Abstract

**Background:**

Oxidative stress contributes significantly to the pathogenesis of acute coronary syndrome (ACS), with neutrophils playing a pivotal role in mediating vascular injury through reactive oxygen species and myeloperoxidase (MPO)-derived oxidants. We aimed to assess the oxidative stress markers and antioxidant enzyme activities in neutrophils from ACS patients.

**Methods:**

Neutrophils were isolated from 77 ACS patients and 33 control subjects. Oxidative stress was evaluated by measuring conjugated dienes, hydroperoxides, and chloramines. Antioxidant status was assessed via non-protein and total thiol levels, and enzymatic activities of superoxide dismutase (SOD), catalase, glutathione peroxidase (GPx-1), and glutathione reductase (GR). MPO peroxidase and chlorinating activities were also quantified.

**Results:**

ACS patients exhibited significantly elevated levels of conjugated dienes (p&lt; 0.005), chloramines (p&lt; 0.005), thiols (p&lt; 0.05), SOD activity (p= 0.01), and MPO chlorinating activity (p&lt; 0.05). No significant differences were observed in catalase, GPx-1, GR, or MPO peroxidase activity. Significant correlations were found between lipid peroxidation markers and antioxidant parameters, particularly SOD and MPO chlorinating activity. Multiple regression identified SOD and chloramines as independent predictors of lipid peroxidation.

**Conclusions:**

Neutrophils from ACS patients display a disrupted redox balance characterised by enhanced lipid peroxidation, increased thiol content, and selective activation of MPO chlorination. These results underscore the relevance of neutrophil-derived redox markers as potential diagnostic tools and treatment targets in ACS.

## Abstract (sr)

**Uvod:**

Oksidativni stres ima ključnu ulogu u patogenezi akutnog koronarnog sindroma (AKS), a neutrofili doprinose oštećenju krvnih sudova preko produkcije reaktivnih kiseoničnih radikala i oksidanasa zavisnih od mijeloperoksidaze (MPO). Cilj istraživanja je bio da se ispitaju promene markera oksidativnog stresa i aktivnosti antioksidativnih enzima u neutrofilima pacijenata sa AKS u poređenju sa kontrolnim subjektima.

**Metode:**

Neutrofili su izolovani iz krvi 77 pacijenata sa AKS i 33 kontrolna subjekta. Oksidativni stres je procenjen merenjem konjugovanih diena, hidroperoksida i hloramina. Antioksidativni status je ocenjen određivanjem nivoa neproteinskih i ukupnih tiolnih grupa, kao i aktivnosti superoksid dismutaze (SOD), katalaze, glutation peroksidaze (GPx-1) i glutation reduktaze (GR). Takođe su određivane peroksidazna i hlorinišuća aktivnost MPO.

**Rezultati:**

U grupi pacijenata sa AKS zabeležene su značajno više koncentracije konjugovanih diena (p&lt; 0,005), hloramina (p&lt; 0,005), tiolnih grupa (p&lt;0,05), aktivnosti SOD (p= 0,05) i hlorinisuće aktivnosti MPO (p&lt;0,05). Nisu nađene značajne razlike u aktivnostima katalaze, GPx-1, GR i peroksidazne aktivnosti MPO. Značajne korelacije su uočene između markera lipidne peroksidacije i antioksidativnih parametara, naročito SOD i MPO hlorinisuće aktivnosti. Višestruka regresiona analiza je identifikovala SOD i hloramine kao nezavisne prediktore lipidne peroksidacije.

**Zaključak:**

Neutrofili pacijenata sa AKS pokazuju izražen redoks disbalans, karakterisan pojačanom lipidnom peroksidacijom, povećanim sadržajem tiola i povećanom hlorinisućom aktivnošću MPO. Ovi nalazi ukazuju na potencijal redoks parametara neutrofila kao biomarkera i terapijskih meta u AKS.

## Introduction

Acute coronary syndrome (ACS) comprises a spectrum of clinical conditions characterised by a sudden reduction in coronary blood flow, encompassing unstable angina pectoris, non-ST-segment elevation myocardial infarction, and ST-segment elevation myocardial infarction. While traditionally attributed to hemodynamic and thrombotic mechanisms, emerging evidence underscores a pivotal role of inflammation and oxidative stress in the pathogenesis and progression of ACS [1][2][3]

As the predominant leukocytes in human blood, neutrophils are key players in the innate immune response and have been increasingly implicated in atherosclerotic plaque destabilisation and myocardial injury [1][4][5]. When stimulated, neutrophils produce reactive oxygen species (ROS), myeloperoxidase (MPO), and proteases that modulate vascular function, contributing to endothelial dysfunction and amplifying local inflammation [6][7][8]. MPO, in particular, generates potent oxidants such as hypochlorous acid, contributing to oxidative tissue damage and lipid peroxidation [9].

The oxidative-antioxidant balance in neutrophils is maintained by enzymatic antioxidants - including superoxide dismutase (SOD), catalase (CAT), glutathione peroxidase (GPx), and glutathione reductase (GR) - which collectively detoxify ROS and preserve redox homeostasis [10][11]. Disruption of this balance results in oxidative stress, a key contributor to the development and progression of ACS.

Oxidative stress biomarkers - such as conjugated dienes, lipid hydroperoxides, chloramines, and thiol levels - serve as indicators of redox imbalance and correlate with disease severity [12][13][14]. Despite accumulating evidence, data on the oxidative profile of neutrophils in ACS remain limited. A detailed assessment of neutrophil redox status could yield novel biomarkers and therapeutic targets.

This study aimed to evaluate the activity of key antioxidant enzymes and the levels of oxidative stress markers in neutrophil lysates from patients with ACS, compared to healthy controls.

## Materials and methods

### Patients

Patients admitted to the Coronary Unit of the Department of Internal Medicine at the Health Centre in Kosovska Mitrovica with chest pain and suspected acute coronary syndrome (ACS) were enrolled. Diagnosis was established according to current clinical guidelines [15][16][17][18], with the final diagnosis determined at discharge. Patients diagnosed with acute myocardial infarction (with or without ST-segment elevation) or unstable angina pectoris (Braunwald classification 111B) were included in the ACS group. The control group consisted of age- and sex-matched healthy volunteer blood donors with no history of coronary heart disease. The study was conducted in accordance with the ethical principles of the Declaration of Helsinki (1975) and approved by the institutional ethics committee. All participants provided written informed consent.

### Methods

Upon admission, 6 mL of venous blood was collected from each subject in EDTA-containing tubes. Biochemical analyses were conducted at the Institute of Biochemistry, Faculty of Medicine, Kosovska Mitrovica.

Neutrophils were isolated using a density gradient centrifugation method described by Boyum [19][20], employing Histopaque-1119 and Histopaque-1077 reagents. Briefly, 3 mL each of Histopaque-1119 and Histopaque-1077 were layered sequentially in a centrifuge tube, followed by 6 mL of whole blood. Centrifugation at 700xg yielded distinct layers: plasma, mononuclear cells, Histopaque-1077, granulocytes, Histopaque-1119, and erythrocytes. The granulocyte layer was carefully aspirated, suspended in phosphate-buffered saline (PBS; pH 7.4), and centrifuged at 200 xg. The washing step was repeated twice. In cases of erythrocyte contamination, a hypotonic NaCl solution (0.033 mol/L) was applied.

Neutrophils were counted using a hemocytometer and a haematology analyser. The purity exceeded 95%, and cell viability was assessed using Trypan blue exclusion. Cells were lysed via two freeze-thaw cycles in the presence of 0.2% Triton X-100 (1:10, v/v) to enhance protein solubilisation. Lysis was confirmed microscopically. The protein concentration of the lysate was standardised to approximately 0.5 g/L across all samples.

Protein concentration in the neutrophil lysates was determined using the Lowry method, as modified by Reider [21], with human serum albumin (1.0 g/L) as the standard.

The concentration of conjugated dienes in neutrophil lysates was measured spectrophotometrically and expressed in μmol/g protein [22][23]. Total hydroperoxide levels, or Fe^2^-induced malondialdehyde equivalents, were quantified using a thiobarbituric acid reaction following Fe^2^ catalysis [14][24]. Results were expressed as pmol/g protein lysate.

The peroxidase activity of MPO (EC 1.11.1.7) was assessed using the Trinder kinetic reaction in a 4-aminoantipyrine/phenol system, with sodium azide added to inhibit catalase peroxidase activity [25]. Results were expressed as units per gram of protein (U/g protein). The chlorinating activity of MPO was determined spectrophotometrically [26]. The assay included hydrogen peroxide, taurine, and 2-nitrobenzoic acid (TNB) at pH 7.4 and 25°C. MPO-generated hypochlorous acid reacts with taurine to produce taurine chloramine, which oxidises TNB to 5,5'-dithiobis-2-nitrobenzoic acid (DTNB), detected at 412 nm. One unit of MPO activity is defined as the enzyme quantity required to produce 1 nmol/L taurine chloramine under the specified conditions. Chlorinating activity was expressed as kilounits per gram of neutrophil protein (kU/g protein). Chloramine concentration was determined using the same assay system as for MPO chlorinating activity [26], by measuring TNB oxidation to DTNB directly in the sample at 412 nm. Values were expressed as μmol/g protein in neutrophil lysate.

The concentration of total non-protein thiol compounds was measured using the Ellman reagent (DTNB) after protein precipitation with perchloric acid [27]. This assay, based on the formation of TNB, was applied using the method of Sedlak and Lindsay [28]. Results were expressed in pmol/g protein. Total thiol concentrations were also assessed using DTNB [27][28], without deproteinisation. The resulting absorbance of 5-thio-2-nitrobenzoic acid was used to quantify total thiol groups, expressed in μmol/g protein.

SOD activity (EC 1.15.1.1) was measured spectrophotometrically according to Misra and Fridovich [29]. This method quantifies the inhibition of adrenochrome formation from adrenaline autoxidation. Absorbance was recorded at 480 nm, and activity expressed as kU/g protein. GPx-1 activity (EC 1.11.1.9) was measured using hydrogen peroxide (0.1%) as substrate, following the method of Chiu et al. [30]. Absorbance was recorded at 412 nm and activity expressed as U/g protein. Catalase activity (EC 1.11.1.6) was determined colorimetrically as per Korolyuk and Goth [31][32], and reported as kU/g protein. GR activity (EC 1.8.1.7) was measured via the nicotinamide adenine dinucleotide phosphate (NADPH)-dependent reduction of oxidised glutathione (GSSG), following Glatzle et al. [33]. The decline in absorbance at 340 nm reflected GR activity, expressed as U/g protein [34][35].

### Statistical analysis

All data were statistically analysed using MedCalc 12.3.0.0 (MedCalc Software, Belgium). Measures of central tendency and variability were calculated. The Kolmogorov-Smirnov test assessed normality. For normally distributed continuous variables, results were presented as mean ± standard deviation (SD), and Student's t-test was used. For non-normally distributed variables, results were expressed as median and 95% confidence interval (CI), and analysed using the Mann-Whitney U test. Categorical data were expressed as counts and percentages. The χ^2^ test or Fisher's exact test (when expected frequencies were <20) was applied for comparisons. Correlation was assessed using Pearson's coefficient (r) with p-values. Linear and multiple regression analyses were performed. A p-value of <0.05 was considered statistically significant.

## Results

The age and gender distribution of both groups is summarised in Table 1. No statistically significant difference was found in the mean age between the acute coronary syndrome (ACS) group and the control group (t=0.97, df=108, p = 0.334). Similarly, gender distribution did not differ significantly between groups (χ^2^=0.087, df=1, p=0.767), indicating adequate matching. Table 1 presents age comparisons by gender within each group. In both the ACS and control groups, female participants were significantly older than their male counterparts (p<0.05).

**Table 1:** Age and gender distribution.

Group	Gender	Age (years), mean ± SD	n	%
Acute coronary syndrome	Male	61.5±11.1	46	59.7%
	Female	66.4±9.9	31	40.3%
**Total**		**63.5±10.8**	**77**	**100%**
Controls	Male	61.0±13.2	18	54.5%
	Female	71.7±11.0	15	45.5%
**Total**		65.8±13.2	**33**	**100%**

The concentrations of conjugated dienes, total hydroperoxides, and chloramines in neutrophil lysates are presented in Table 2. Values are expressed in μmol/g protein and reported as median (interquartile range). Conjugated dienes were significantly higher in the ACS group compared to controls. Total hydroperoxides did not differ significantly between groups. Chloramine levels were elevated in the ACS group.

**Table 2:** Oxidative modification products in neutrophils. Data are presented as median (interquartile range, Q1-Q3).

Parameters	Acute coronary syndrome patients	Control group	p-value
Conjugated dienes(mmol/g protein)	416 (378.6-587.6)	369 (342.5-385.6)	<0.005
Hydroperoxides(mmol/g protein)	2.6 (1.9-3.5)	2.8 (2.4-3.7)	0.3871
Chloramines(mmol/g protein)	48.6 (36.3-68.2)	33.3 (28.6-40.9)	<0.005

The concentration of non-protein thiol groups and total thiol groups in neutrophil lysate was expressed in μmol/g protein lysate. The activity of superoxide dismutase (kU/g protein), catalase (kU/g protein), glutathione peroxidase (U/g) and glutathione reductase (U/g) in neutrophils is presented in Table 3. Values are reported as median (interquartile range). Non-protein thiol groups were higher in the ACS group vs. controls. Total thiol groups were significantly higher in the ACS group compared to controls. Superoxide dismutase activity was higher in the ACS group compared to controls. Catalase, glutathione peroxidase-1 and glutathione reductase did not differ significantly between groups.

**Table 3:** Antioxidative status in neutrophils. Data are presented as median (interquartile range, Q1-Q3).

Parameters	Acute coronary syndrome patients	Control group	p-value
Non-protein thiols (mmol/g protein)	23.2 (14.6-32.0)	11.2 (8.9-17.4)	<0.005
Total thiols (mmol/g protein)	108.5 (69.1-145.4)	63.7 (29.8-138.3)	0.0308
Superoxide dismutase (kU/g protein)	88.9 (51.8-133.5)	56.3 (19.4-101.0)	0.0105
Catalase (kU/g protein)	31.5 (10.6-52.9)	29.2 (10.5-52.5)	0.648
Glutathione peroxidase-1 (U/g protein)	11.6 (5.2-35.9)	26.7 (11.8-38.0)	0.0905
Glutathione reductase (U/g protein)	13.7 (5.9-28.9)	13.8 (4.8-37.5)	0.4261

Values of the peroxidase (U/g protein) and chlorinating activity (kU/g protein) of myeloperoxidase are reported as median (interquartile range). The results are shown in Table 4. Chlorinating activity of MPO was significantly higher in the ACS group compared to controls. Peroxidase activity of MPO did not differ significantly between groups.

**Table 4:** Catalytic activity of myeloperoxidase in neutrophils. Data are presented as median (interquartile range, Q1-Q3).

Parameters	Acute coronary syndrome patients	Control group	p-value
Peroxidase activity (U/g protein)	16 (5.5-25.6)	18.3 (4.0-30.1)	0.9948
Chlorinating activity (kU/g protein)	83.3 (65.4-118.5)	65.5 (47.2-89.2)	0.0045

The measure of interdependence of logarithmically transformed values of variables from neutrophil lysates was examined. Logarithmic transformation was performed to establish a normal distribution of the data and to calculate the Pearson correlation coefficient. The analysis was conducted in a group of patients with acute coronary syndrome. Significant positive correlations (p<0.05) were observed (Table 5) between the conjugated dienes and: non-protein thiols, total thiols, superoxide dismutase (SOD), myeloperoxidase (MPO), chlorinating activity, and chloramines. Further, positive correlations (p<0.05) were observed between chloramines and non-protein thiols, total thiols, superoxide dismutase, glutathione reductase (GRed), and MPO chlorinating activity. Positive correlations (p<0.05) were observed between MPO chlorinating activity and: non-protein thiols, total thiols, superoxide dismutase, and glutathione reductase.

**Table 5:** Correlation between parameters in neutrophils in acute coronary syndrome. Statistically significant Pearson correlation coefficients (p<0.05) are presented; Abbreviations: MPO-myeloperoxidase, SOD-superoxide dismutase, GR-glutathione reductase.

Parameters<br>(logarithmically<br>transformed)	Non-protein<br>thiols<br>(mmol/g<br>protein)	Total thiols<br>(mmol/g<br>protein)	SOD<br>(kU/g<br>protein)	MPO<br>chlorinating<br>activity<br>(kU/g protein)	Chloramines<br>(mmol/g<br>protein)	GR<br>(U/g protein)
Conjugated dienes<br>(mmol/g protein)	0.343	0.332	0.595	0.458	0.486	
Chloramines<br>(mmol/g protein)	0.348	0.398	0.43	0.773		0.229
MPO chlorinating<br>activity<br>(kU/g protein)	0.315	0.359	0.544		0.773	0.322

Multiple linear regression was performed to identify independent predictors of oxidative stress markers. Variables with prior significant correlations (Pearson's r between 0.3 and 0.7) were included.

For log-transformed conjugated dienes (Table 6) , the final regression model included log SOD activity and log chloramine levels as significant predictors (p<0.001). The model explained 40.36% of the total variance (adjusted R^2^=0.4036; F=26.72, p<0.001).

**Table 6:** Linear and multiple regression with log concentration of conjugated dienes (mmol/g protein) in neutrophil lysate in the group of patients with acute coronary syndrome. Abbreviations: B-unstandardized coefficient (»slope«), SE-standard error, β-standardized coefficient, log-values of independent variables are logarithmically transformed. MPO- myeloperoxidase.

Independent<br>variable	Linear regression			Multiple regression		
	B	SE	p	β	SE	p
log superoxide<br>dismutase<br>(kU/g protein)	0.2640	0.04117	<0.0001	0.2101	0.04353	<0.0001
log chloramines<br>(μmol/g protein)	0.4287	0.08894	<0.0001	0.2492	0.08650	0.0052
log non-protein thiols<br>(μmol/g protein)	0.1397	0.04422	0.0023			
log total thiols<br>(μmol/g protein)	0.1954	0.06406	0.0032			
log MPO chlorinating <br>activity (kU/g protein)	0.3859	0.08649	<0.0001			

For log-transformed chloramine levels (Table 7) , significant predictors were log conjugated dienes and log total thiols. The model accounted for 28.04% of the variance (adjusted R^2^=0.2804; F=15.81, p<0.001).

**Table 7:** Linear and multiple regression with log chloramine concentration (mmol/g protein) in neutrophil lysate in the group of patients with acute coronary syndrome. Abbreviations: B-unstandardised coefficient (»slope«), SE-standard error, β-standardised coefficient, log-values of independent variables are logarithmically transformed.

Independent variable	Linear regression			Multiple regression		
	B	SE	p	β	SE	p
log conjugated dienes<br>(μmol/g protein)	0.5517	0.1145	<0.0001	0.4517	0.1170	0.0002
log total thiols<br>(μmol/g protein)	0.2655	0.07069	0.0003	0.1772	0.06884	0.0120
log non-protein thiols<br>(μmol/g protein)	0.1608	0.05007	0.0019			
log superoxide dismutase<br>(kU/g protein)	0.2165	0.05246	0.0001			

## Discussion

This study provides a detailed evaluation of oxidative stress markers and antioxidant defences in neutrophils from patients with acute coronary syndrome (ACS). The results reinforce the hypothesis that neutrophil-derived oxidative imbalance contributes significantly to the pathophysiology of ACS.

Neutrophils, beyond their inflammatory role, are key sources of reactive oxygen species (ROS) and chlorinating agents, particularly through NADPH oxidase and myeloperoxidase (MPO) activation [4][5]. The observed increase in superoxide dismutase (SOD) activity suggests enhanced conversion of superoxide to hydrogen peroxide in neutrophils during ACS, consistent with previous reports of neutrophil priming in ischemic conditions [36].

Interestingly, while SOD activity was elevated, catalase and glutathione peroxidase-1 (GPx-1) levels remained unchanged. This imbalance may result in hydrogen peroxide buildup, driving increased oxidative pressure within the cell. MPO utilises hydrogen peroxide to produce hypochlorous acid (HOCl), and our findings of elevated MPO chlorinating activity support increased oxidative conversion under ischemic stress. The unchanged MPO peroxidase activity may indicate selective activation of its chlorinating function in ACS, as previously documented [6].

The study also revealed significantly increased levels of non-protein and total thiol groups in neutrophils from ACS patients. This could reflect a compensatory response to oxidative stress, or potentially a state of reductive stress - a condition characterised by excessive reducing equivalents such as glutathione. An excess of reducing agents, termed reductive stress, is associated with impaired mitochondrial activity and altered redox-dependent signalling pathways, paradoxically contributing to oxidative damage [37][38]. Increased thiol concentrations without parallel upregulation of detoxifying enzymes may exacerbate redox imbalance and support prolonged neutrophil viability, a phenomenon noted in ACS [39].

Lipid peroxidation products, particularly conjugated dienes and chloramines, were significantly elevated, indicating increased oxidative burden in neutrophils. However, the lack of change in total hydroperoxides suggests that oxidative events may be transient or compartmentalised, potentially limited by reduced oxygen availability during ischemia [40].

A key finding is the robust correlation between MPO chlorinating activity, SOD activity, and chloramine levels. This indicates a sequential oxidative cascade: SOD generates hydrogen peroxide, which fuels MPO activity and subsequent chlorination of biomolecules. This pathway is thought to contribute to endothelial dysfunction and the destabilisation of atherosclerotic plaques during ACS [9].

Furthermore, the positive association between MPO activity and thiol concentrations supports the idea that reduced thiol compounds, such as glutathione, enhance MPO chlorination by maintaining redox potential for HOCl production. This is consistent with prior studies on thiol-mediated regulation of MPO activity [12][41].

Overall, these findings suggest that oxidative and reductive stresses coexist and interact within neutrophils during ACS. While ROS excess is well recognised in ACS pathology, the role of excessive reducing equivalents in sustaining oxidative enzyme activity and inflammatory signalling warrants further investigation.

This study has several limitations. First, its crosssectional design precludes causal inference between redox changes and clinical outcomes. Second, the absence of NADPH oxidase measurements limits the complete characterisation of ROS sources. Third, results were normalised to protein concentration, which may not account for cell-to-cell variability. Fourth, only a single time point was analysed, limiting insight into the dynamic evolution of redox markers. Lastly, potential confounders such as comorbidities, medications, and lifestyle factors were not fully controlled and may influence oxidative stress parameters.

## Conclusion

This study demonstrates that neutrophils from patients with acute coronary syndrome (ACS) exhibit a profoundly altered redox state, marked by enhanced lipid peroxidation, elevated thiol content, and increased chlorinating activity of myeloperoxidase (MPO). These changes reflect a dysregulated interplay between oxidative and reductive stress mechanisms.

The findings highlight neutrophil redox dysregulation as a critical component of ACS pathophysiology. Elevated superoxide dismutase activity alongside unaltered catalase and glutathione peroxidase levels suggests a disrupted antioxidant response, possibly promoting hydrogen peroxide accumulation and MPO-driven oxidative damage. The concurrent rise in thiol levels may represent a compensatory but potentially maladaptive response, contributing to prolonged neutrophil activity and tissue injury.

Overall, these results support the utility of neutrophil redox markers - particularly MPO chlorinating activity and thiol status - as potential diagnostic or prognostic indicators in ACS. Moreover, targeting neutrophil redox imbalance could offer novel therapeutic avenues to mitigate ischemic injury and inflammation in acute coronary events.

## Dodatak

### Conflict of interest statement

All the authors declare that they have no conflict of interest in this work.

## References

[1] Libby P (2002). Inflammation in atherosclerosis. Arterioscler Thromb Vasc Biol.

[2] Hansson G K (2005). Inflammation, Atherosclerosis, and Coronary Artery Disease. N Engl J Med.

[3] Ridker P M, Hennekens C H, Buring J E, Rifai N (2000). C-Reactive Protein and Other Markers of Inflammation in the Prediction of Cardiovascular Disease in Women. N Engl J Med.

[4] Soehnlein O (2012). Multiple roles for neutrophils in atherosclerosis. Circ Res.

[5] Quillard T, Araújo H A, Franck G, Shvartz E, Sukhova G, Libby P (2015). TLR2 and neutrophils potentiate endothelial stress, apoptosis and detachment: implications for superficial erosion. Eur Heart J.

[6] Nicholls S J, Hazen S L (2005). Myeloperoxidase and cardiovascular disease. Arterioscler Thromb Vasc Biol.

[7] Lubrano V, Pingitore A, Traghella I, Storti S, Parri S, Berti S, et al (2019). Emerging Biomarkers of Oxidative Stress in Acute and Stable Coronary Artery Disease: Levels and Determinants. Antioxidants.

[8] Zhang R, Brennan M L, Fu X, Aviles R J, Pearce G L, Penn M S, et al (2001). Association Between Myeloperoxidase Levels and Risk of Coronary Artery Disease. JAMA.

[9] Weiss S J (1989). Tissue destruction by neutrophils. N Engl J Med.

[10] Halliwell B, Gutteridge J M C (2015). Free Radicals in Biology and Medicine. 5th ed.

[11] Erel O (2004). A novel automated direct measurement method for total antioxidant capacity using a new generation, more stable ABTS radical cation. Clin Biochem.

[12] Peskin A V, Winterbourn C C (2003). Histamine chloramine reactivity with thiol compounds, ascorbate, and methionine and with intracellular glutathione. Free Rad Biol Med.

[13] Karanikolic V, Bakic M, Gluscevic S, Mercantepe F, Klisic A (2025). Cardiovascular risk in patients with a relationship with oxidative stress and dyslipidemia. J Med Biochem.

[14] Asakawa T, Matsushita S (1979). Thiobarbituric acid test for detecting lipid peroxides. Lipids.

[15] Bertrand M E, Simoons M L, Fox K A, Wallentin L C, Hamm C W, McFadden E, et al (2002). Task Force on the Management of Acute Coronary Syndromes of the European Society of Cardiology. Management of acute coronary syndromes in patients presenting without persistent ST-segment elevation. Eur Heart J.

[16] Van de Werf F, Ardissino D, Betriu A, Cokkinos D V, Falk E, Fox K A, et al (2003). Task Force on the Management of Acute Myocardial Infarction of the European Society of Cardiology. Management of acute myocardial infarction in patients presenting with ST-segment elevation. Eur Heart J.

[17] Hamm C W, Braunwald E (2000). A Classification of Unstable Angina Revisited. Circulation.

[18] 18. *** (2000). Myocardial infarction redefined-A consensus document of The Joint European Society of Cardiology/American College of Cardiology Committee for the Redefinition of Myocardial Infarction. Eur Heart J.

[19] Boyum A (1968). Separation of leukocytes from blood and bone marrow. Scand J Clin Lab Invest Suppl.

[20] English D, Andersen B R (1974). Single-step separation of red blood cells, granulocytes and mononuclear leukocytes on discontinuous density gradients of Ficoll-Hypaque. J Immunol Methods.

[21] Lowry O H, Rosebrough N J, Farr A L, Randall R J (1951). Protein measurement with the Folin phenol reagent. J Biol Chem.

[22] Recknagel R O, Glende E A (1984). Spectrophotometric detection of lipid conjugated dienes. Methods Enzymol.

[23] Tappel A L, Pryor W A (1980). Free Radicals in Biology.

[24] Babizhayev M A, Costa B E (1994). Lipid peroxide and reactive oxygen species generating systems of the crystalline lens. Biochim Biophys Acta.

[25] Metcalf J A, Gallin J I, Nauseef W M, Root R K, Metcalf J A (1986). Laboratory Manual of Neutrophil Function.

[26] Dypbukt J M, Bishop C, Brooks W M, Thong B, Eriksson H, Kettle A J (2005). A sensitive and selective assay for chloramine production by myeloperoxidase. Free Rad Biol Med.

[27] Dacie S J V, Lewis S M, Dacie S J V (1984). Practical Haematology, 6th ed.

[28] Sedlak J, Lindsay R H (1968). Estimation of total, protein-bound, and non-protein sulfhydryl groups in tissue with Ellman's reagent. Anal Biochem.

[29] Misra H P, Fridovich I (1972). The Role of Superoxide Anion in the Autoxidation of Epinephrine and a Simple Assay for Superoxide Dismutase. J Biol Chem.

[30] Chiu D T, Stults F H, Tappel A L (1976). Purification and properties of rat lung soluble glutathione peroxidase. Biochim Biophys Acta.

[31] Koroliuk M A, Ivanova L I, Maïorova I G, Tokarev V E (1988). Metod opredeleniia aktivnosti katalazy (A method of determining catalase activity). Lab Delo.

[32] Góth L (1991). A simple method for determination of serum catalase activity and revision of reference range. Clin Chim Acta.

[33] Glatzle D, Vuilleumier J P, Weber F, Decker K (1974). Glutathione reductase test with whole blood, a convenient procedure for the assessment of the riboflavin status in humans. Experientia.

[34] Majkic Singh N (1993). Klinička enzimologija (Clinical enzymology).

[35] Bukowska B (2004). 2,4,5-T and 2,4,5-TCP induce oxidative damage in human erythrocytes: the role of glutathione. Cell Biol Int.

[36] Buffon A, Biasucci L M, Liuzzo G, D'Onofrio G, Crea F, Maseri A (2002). Widespread Coronary Inflammation in Unstable Angina. N Engl J Med.

[37] Zhang H, Limphong P, Pieper J, Liu Q, Rodesch C K, Christians E, et al (2012). Glutathione-dependent reductive stress triggers mitochondrial oxidation and cytotoxicity. FASEB J.

[38] Yu Q, Lee C F, Wang W, Karamanlidis G, Kuroda J, Matsushima S, et al (2014). Elimination of NADPH Oxidase Activity Promotes Reductive Stress and Sensitizes the Heart to Ischemic Injury. J Am Heart Assoc.

[39] Garlichs C D, Eskafi S, Cicha I, Schmeisser A, Walzog B, Raaz D, et al (2004). Delay of neutrophil apoptosis in acute coronary syndromes. J Leukoc Biol.

[40] Wright A, Bubb W A, Hawkins C L, Davies M J (2002). Singlet oxygen-mediated protein oxidation: evidence for the formation of reactive side chain peroxides on tyrosine residues. Photochem Photobiol.

[41] Marquez L A, Dunford H B, Van Wart H (1990). Kinetic studies on the reaction of compound II of myeloperoxidase with ascorbic acid. Role of ascorbic acid in myeloperoxidase function. J Biol Chem.

